# p150^glued^-Associated Disorders Are Caused by Activation of Intrinsic Apoptotic Pathway

**DOI:** 10.1371/journal.pone.0094645

**Published:** 2014-04-10

**Authors:** Kei-Ichi Ishikawa, Shinji Saiki, Norihiko Furuya, Daisuke Yamada, Yoko Imamichi, Yuanzhe Li, Sumihiro Kawajiri, Hironori Sasaki, Masato Koike, Yoshio Tsuboi, Nobutaka Hattori

**Affiliations:** 1 Department of Neurology, Juntendo University School of Medicine, Tokyo, Japan; 2 Department of Research and Therapeutics for Movement Disorders, Juntendo University School of Medicine, Tokyo, Japan; 3 Department of Cell Biology and Neuroscience, Juntendo University School of Medicine, Tokyo, Japan; 4 Department of Neurology, Fukuoka University School of Medicine, Fukuoka, Japan; Hokkaido University, Japan

## Abstract

Mutations in p150^glued^ cause hereditary motor neuropathy with vocal cord paralysis (HMN7B) and Perry syndrome (PS). Here we show that both overexpression of p150^glued^ mutants and knockdown of endogenous p150^glued^ induce apoptosis. Overexpression of a p150^glued^ plasmid containing either a HMN7B or PS mutation resulted in cytoplasmic p150^glued^-positive aggregates and was associated with cell death. Cells containing mutant p150^glued^ aggregates underwent apoptosis that was characterized by an increase in cleaved caspase-3- or Annexin V-positive cells and was attenuated by both zVAD-fmk (a pan-caspase inhibitor) application and caspase-3 siRNA knockdown. In addition, overexpression of mutant p150^glued^ decreased mitochondrial membrane potentials and increased levels of translocase of the mitochondrial outer membrane (Tom20) protein, indicating accumulation of damaged mitochondria. Importantly, siRNA knockdown of endogenous p150^glued^ independently induced apoptosis via caspase-8 activation and was not associated with mitochondrial morphological changes. Simultaneous knockdown of endogenous p150^glued^ and overexpression of mutant p150^glued^ had additive apoptosis induction effects. These findings suggest that both p150^glued^ gain-of-toxic-function and loss-of-physiological-function can cause apoptosis and may underlie the pathogenesis of p150^glued^-associated disorders.

## Introduction

The dynactin subunit p150^glued^ is encoded by the *DCTN1* gene. Mutations in this gene have been detected in patients with slowly progressive autosomal dominant distal hereditary motor neuropathy with vocal cord paralysis (HMN7B) and autosomal dominant Perry syndrome (PS), the latter of which is characterized by rapidly progressive, devastating neurodegeneration of dopaminergic neurons in the substantia nigra [1], [2].

Dynactin has various molecular functions including minus-end vesicular transport, protein degradation, and cell division. p150^glued^ is the largest polypeptide of the dynactin complex, and it binds directly to microtubules and to cytoplasmic dynein. Disruption of the p150^glued^ CAP-Gly domain in neurons causes insufficient retrograde axonal transport [3], [4]. Transgenic mice expressing p150^glued^ with a G59S mutation develop progressive degeneration of motor neurons similar to that seen in amyotrophic lateral sclerosis [5]–[7]. The mutated p150^glued^ polypeptide that causes PS is unable to bind to microtubules and forms intracytoplasmic aggregates. These aggregates include abnormally accumulated mitochondria [11]. Despite these findings, it is unclear whether decreased levels of endogenous p150^glued^ or increased levels of the mutant form dominantly contribute to the neurodegeneration seen in PS.

Here we report that knockdown of endogenous p150^glued^ and overexpression of p150^glued^ with pathogenic HMN7B or PS mutations independently induced apoptosis. However, only overexpression of mutant forms of p150^glued^ induced intracytoplasmic p150^glued^-aggregates and accumulation of damaged mitochondria, resulting in intrinsic apoptosis induction. Importantly, mutant p150^glued^ overexpression with endogenous p150^glued^ knockdown showed additive effects on apoptosis induction, suggesting that both a gain- and loss-of-function contribute to the disease pathogenesis.

## Results

### Cells overexpressing various p150^glued^ mutants produce cytoplasmic aggregates

**Figure 1:**
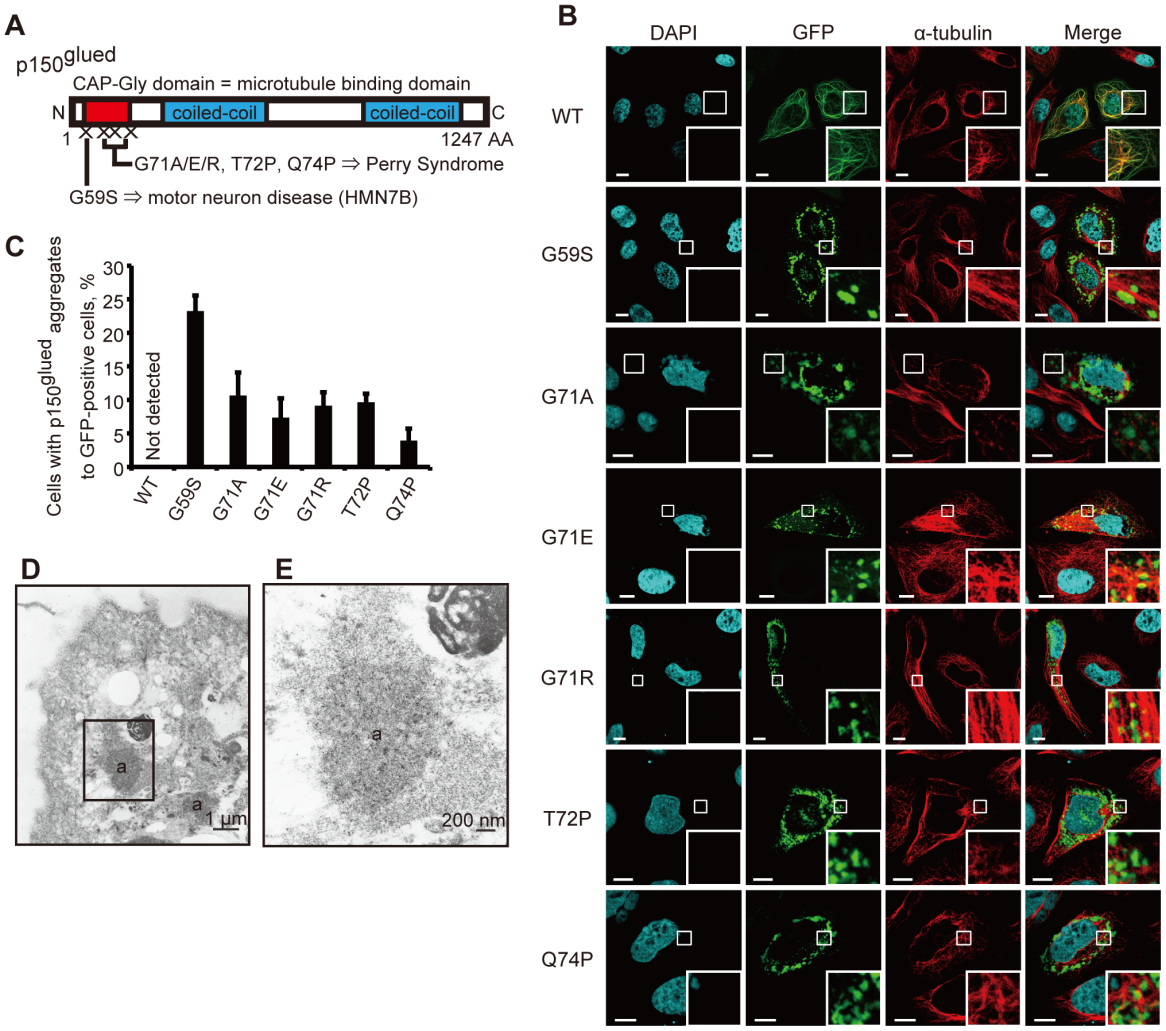
Disease-associated p150^glued^ mutant proteins form aggregates. (A) Schematic of the p150^glued^ subunit of dynactin. (B) HeLa cells transfected with GFP-tagged wild-type or mutant p150^glued^ were fixed and stained with an antibody against α-tubulin (red) after 24 h and analyzed using confocal microscopy. Insets show higher magnification of the boxed areas. Bars, 10 μm. (C) The percentages of GFP-positive cells that contained aggregates are shown. The error bar indicates each standard deviation. Statistics are from three independent experiments. (D, E) Electron micrographic examination of HeLa cells transfected with GFP-tagged G59S p150^glued^ and immunolabeled with an antibody against GFP. (E) High magnification of the boxed area shown in (D). Intracytoplasmic aggregates (a) are labeled.

To investigate the effects of overexpression of mutant p150^glued^, we first generated plasmid DNAs encoding GFP- or 3xFLAG-tagged wild-type (WT) and mutant p150^glued^ with each pathogenic mutation: G59S, which causes HMN7B, and G71A, G71E, G71R, T72P, or Q74P, which cause PS. All of these mutations are within the p150^glued^ CAP-Gly microtubule binding domain (Figure 1A).

To determine if a mutation in p150^glued^ affected its intracellular localization, we transfected GFP-tagged WT or mutant p150^glued^ into HeLa cells followed by immunocytochemical analysis. HeLa cells overexpressing GFP-WT p150^glued^ showed complete colocalization with tubulin (Figure 1B). By contrast, those with a pathogenic mutation were diffusely distributed in the cytoplasm and showed no apparent colocalization with tubulin (Figure S1A). Additionally, cytoplasmic, but not nuclear, aggregates were observed in cells with high expression levels of the mutant p150^glued^ plasmids as early as 24 h after transfection, most frequently in the perinuclear region of the cells with G59S p150^glued^ (Figure 1B, C). These findings are consistent with previous reports [8], [11]. Analogous results were detected with the overexpression of 3xFLAG-tagged WT and mutant p150^glued^ in SH-SY5Y (Figure S1B) and HeLa cells (Figure S1C, D). Previous studies examining the overexpression of mutant G59S p150^glued^ showed decreased affinity of the mutant form of p150^glued^ for microtubules, indicating that mutant p150^glued^ dissociated from microtubules and formed aggregates [11].

To confirm the formation of cytoplasmic aggregates, we performed conventional electron microscopy (EM) analysis. High-density aggregates around the nuclei were detected in cells overexpressing G59S or G71R p150^glued^ (Figure S1E). Next, using immuno-EM analysis with anti-GFP antibodies to recognize GFP-tagged mutant p150^glued^, we detected p150^glued^ localized in high density aggregates, particularly in the perinuclear region of the cells overexpressing G59S (Figure 1D, E) or G71R (data not shown) p150^glued^. Unfortunately, because of the fixation technique for immuno-EM, we could not use the same specimens to assess morphological changes in organelles, including mitochondria, in the cells that showed the aggregates.

We next sought to determine the characteristics of the aggregates by immunocytochemistry. The mutant p150^glued^ aggregates were partially positive for endogenous ubiquitin but not for FLAG-tagged TAR DNA-binding protein 43 (TDP-43) (Figure S1F, G). This is consistent with previous reports showing that dynactin subunits p50 and p62 were present in less than 5% of TDP-43-expressing neurons in the globus pallidus of the autopsied brain of a PS patient [8].

### Apoptotic changes occurred in cells with cytoplasmic aggregates

**Figure 2:**
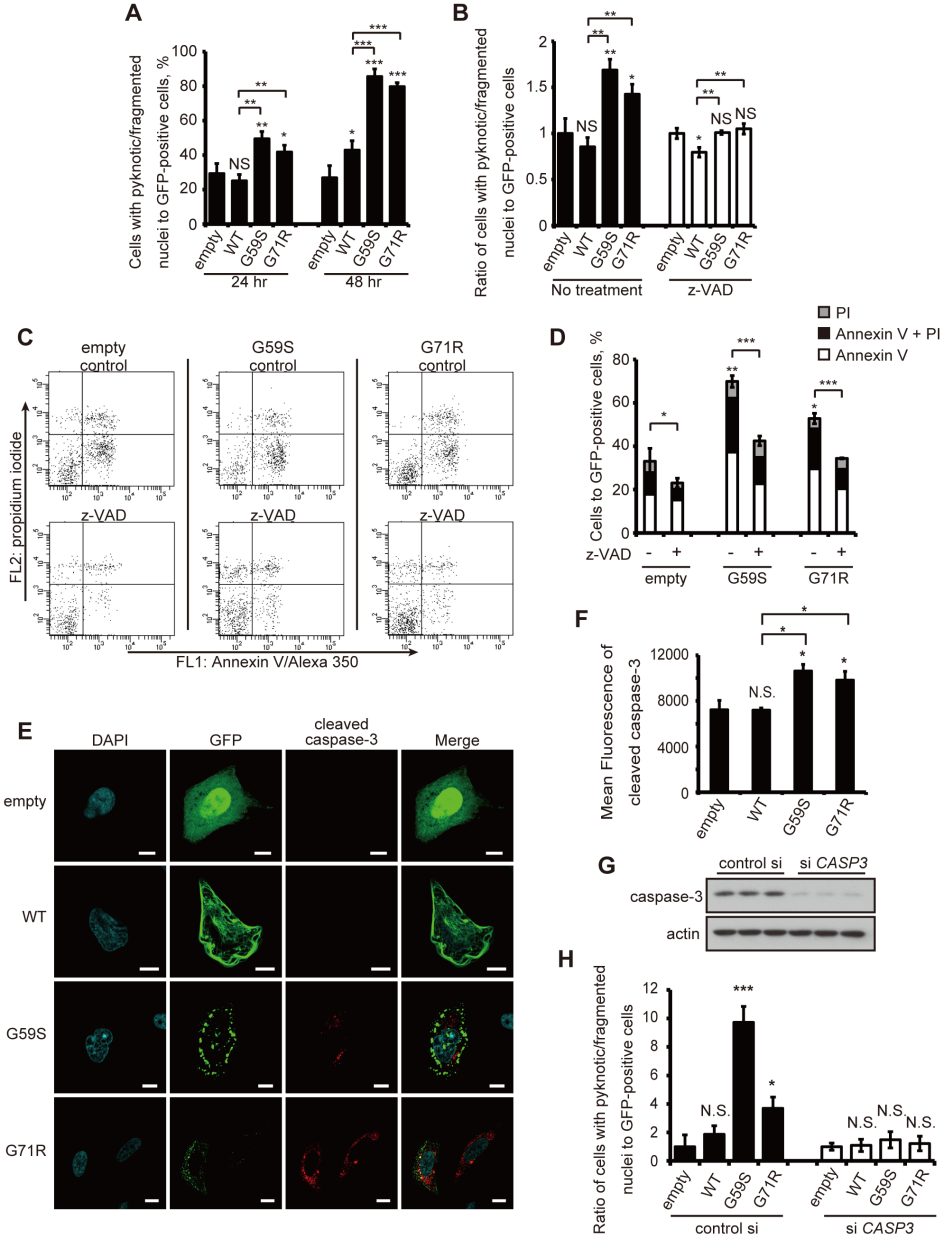
Mutant p150^glued^ proteins activate intrinsic apoptotic pathways. (A) HeLa cells transfected with GFP-empty, GFP-tagged wild-type or mutant (G59S or G71R) p150^glued^ were fixed and stained with DAPI after 24 and 48 h. GFP-positive cells were counted from three independent experiments. The percentage of GFP-positive cells with nuclear abnormalities is shown. (B) Ratio of transfected HeLa cells with nuclear abnormalities after treatment with or without z-VAD (100 μM) for 24 hours. Values are relative to the GFP-empty value, which is set at 1. (C, D) Transfected SH-SY5Y cells after treatment with or without z-VAD (100 μM) for 48 h were stained with Annexin V and PI, and GFP-positive cells were analyzed by flow cytometry. (E) HeLa cells transfected with GFP-tagged wild-type or mutant p150^glued^ were fixed and stained with an antibody against cleaved caspase-3. Bars, 10 μm. (F) Twenty-four hours after transfection, SH-SY5Y cells were fixed and stained with a cleaved caspase-3 antibody. GFP-positive cells were analyzed by flow cytometry and the mean fluorescent intensity was calculated. (G) HeLa cells were transfected with control scrambled siRNA or caspase-3 siRNA for 72 h and immunoblotting analysis was performed to monitor the knockdown efficiency of the caspase-3 siRNA. (H) Twenty-four hours after transfection with control siRNA or caspase-3 siRNA, HeLa cells were transfected with GFP-empty, GFP-tagged wild-type or mutant p150^glued^. Forty-eight hours after transfection, cells were fixed and stained with DAPI, and ratios of GFP-positive cells with nuclear abnormalities were analyzed. Values are relative to the GFP-empty value, which is set at 1. The error bar indicates each standard deviation. Statistics are from three independent experiments: N.S., not significant; *, p<0.05; **, p<0.01; ***, p<0.001.

To elucidate the pathogenesis of the p150^glued^-associated diseases, we focused on the association of cytoplasmic aggregates induced by mutant p150^glued^ overexpression with cell death. We tested the death rate of cells expressing the GFP-tagged p150^glued^, assessed by nuclear morphological changes described in a previous report [12]. The rate of cell death was significantly increased by overexpression of G59S or G71R p150^glued^ both 24 and 48 h after transfection when compared with control cells (Figure 2A).

To examine the characteristics of the cell death induced by G59S or G71R p150^glued^, we performed the same analysis 24 h after transfection with controls cells and cells treated with the pan-caspase inhibitor carbobenzoxy-valyl-alanyl-aspartyl-[O-methyl]-fluoromethylketone (z-VAD-fmk). The rate of cell death induced by overexpression of G59S or G71R p150^glued^ was significantly suppressed by z-VAD (Figure 2B) [13]. In a population of cells selected based on GFP (p150^glued^) intensity, the numbers of early apoptotic cells (annexin V-positive, propidium iodide (PI)-negative) and late apoptotic or necrotic cells (annexin V-positive, PI-positive) were both increased by overexpression of G59S or G71R p150^glued^ (Figure 2C, D). Likewise, z-VAD treatment significantly decreased cell death in both G59S and G71R p150^glued^-overexpressing cells.

To examine the activation of the intrinsic apoptotic pathway, we determined whether or not cells with aggregates were positive for cleaved caspase-3 using fluorescent-activated cell sorting (FACS) and immunocytochemistry analyses. The number of cells positive for both cleaved caspase-3 and GFP (p150^glued^) in cells overexpressing G59S or G71R p150^glued^ was markedly increased compared with control cells (Figure 2E, F). We found that siRNA knockdown against caspase-3 blocked the increase of cell death caused by the overexpression of the mutant p150^glued^ (Figure 2G, H). Next, we wanted to rule out the possibility that extrinsically induced apoptosis via caspase-8 cleavage was causing some or all of the cell death seen in these experiments. Therefore, to exclude this possibility, we examined whether siRNA knockdown of caspase-8 inhibited the apoptosis induced by overexpression of mutant p150^glued^. Knockdown of caspase-8 did not inhibit apoptosis induced by overexpression of G59S p150^glued^ (Figure S2), suggesting that extrinsically induced apoptosis is not the cause of the cell death seen in these cells.

Aggregate formation caused by the G59S mutant led to cell death. Both aggregate formation and cell death are inhibited by overexpression of Hsp70, a molecular chaperone. These findings suggest that mutant p150^glued^ aggregates play an important role in the mechanism of cell death in HMN7B [11]. We conclude that mutant p150^glued^ aggregates cause apoptosis via activation of the intrinsic apoptotic pathway.

### Cells with cytoplasmic aggregates have more mitochondria with reduced mitochondrial membrane potentials

**Figure 3:**
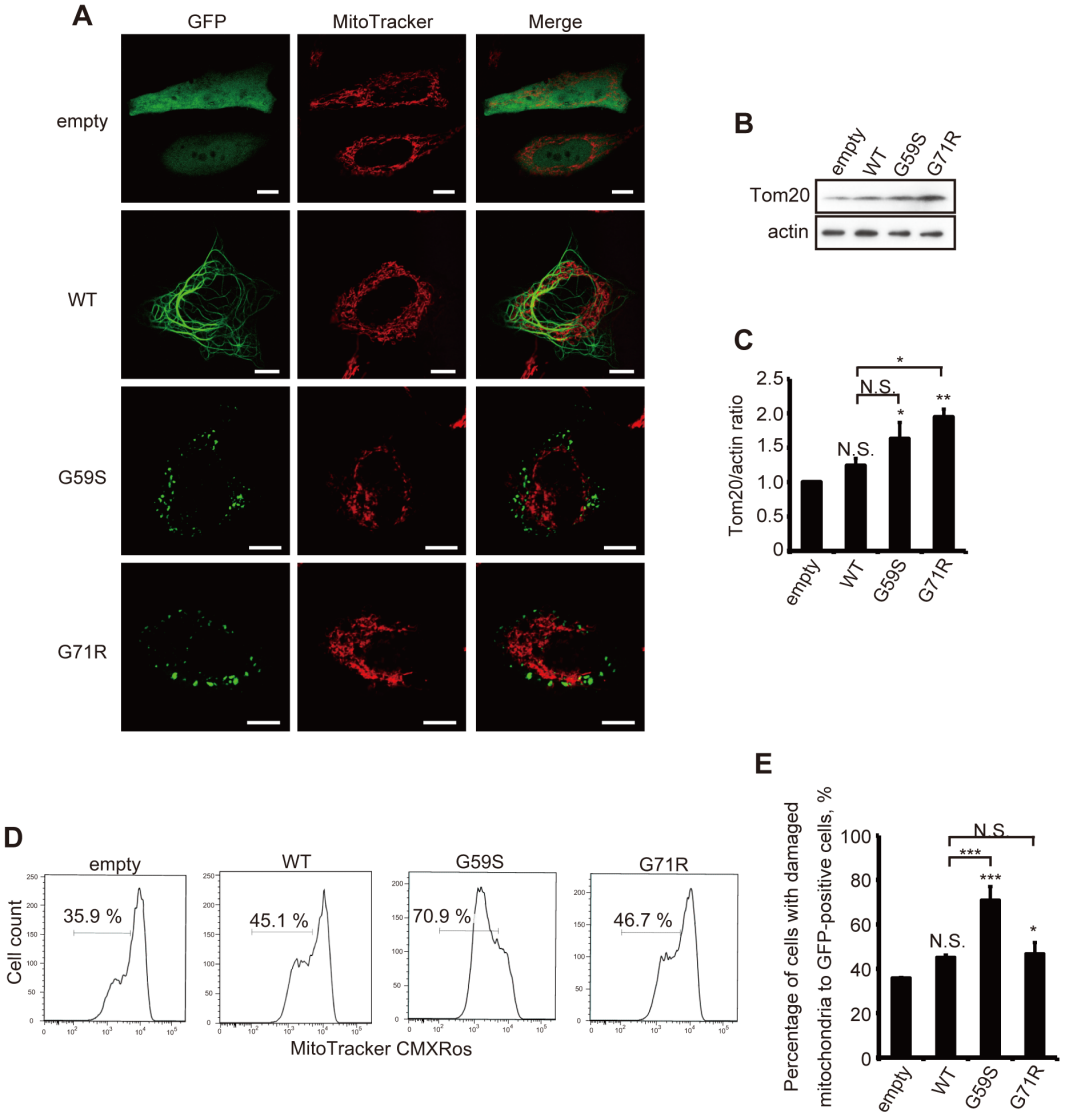
Abnormal mitochondria accumulate in cells overexpressing mutant p150^glued^. (A) HeLa cells transfected with GFP-empty vector, GFP-tagged wild-type or mutant (G59S or G71R) p150^glued^ were incubated with MitoTracker Deep Red (100 nM) for 15 min and analyzed using confocal microscopy. Insets show higher magnification of the boxed areas. Bars, 10 μm. (B, C) Twenty-four hours after transfection, GFP-positive HeLa cells were sorted using flow cytometry and analyzed by immunoblotting with antibodies against TOM20 and actin (B). Densitometry analysis of TOM20 levels relative to actin was performed in three independent experiments (C). (D, E) Twenty-four hours after transfection, HeLa cells were incubated with Mitotracker-Red CMXRos (25 nM) for 15 min, and intracellular fluorescence intensity was measured by flow cytometry. The histograms of MitoTracker-Red CMXRos fluorescence in GFP-positive cells (D) and the percentages of GFP-positive cells with reduced mitochondrial potentials (E) are shown. The error bar indicates each standard deviation. Statistics are from three independent experiments: *, p<0.05; **, p<0.01; ***, p<0.001.

**Figure 4:**
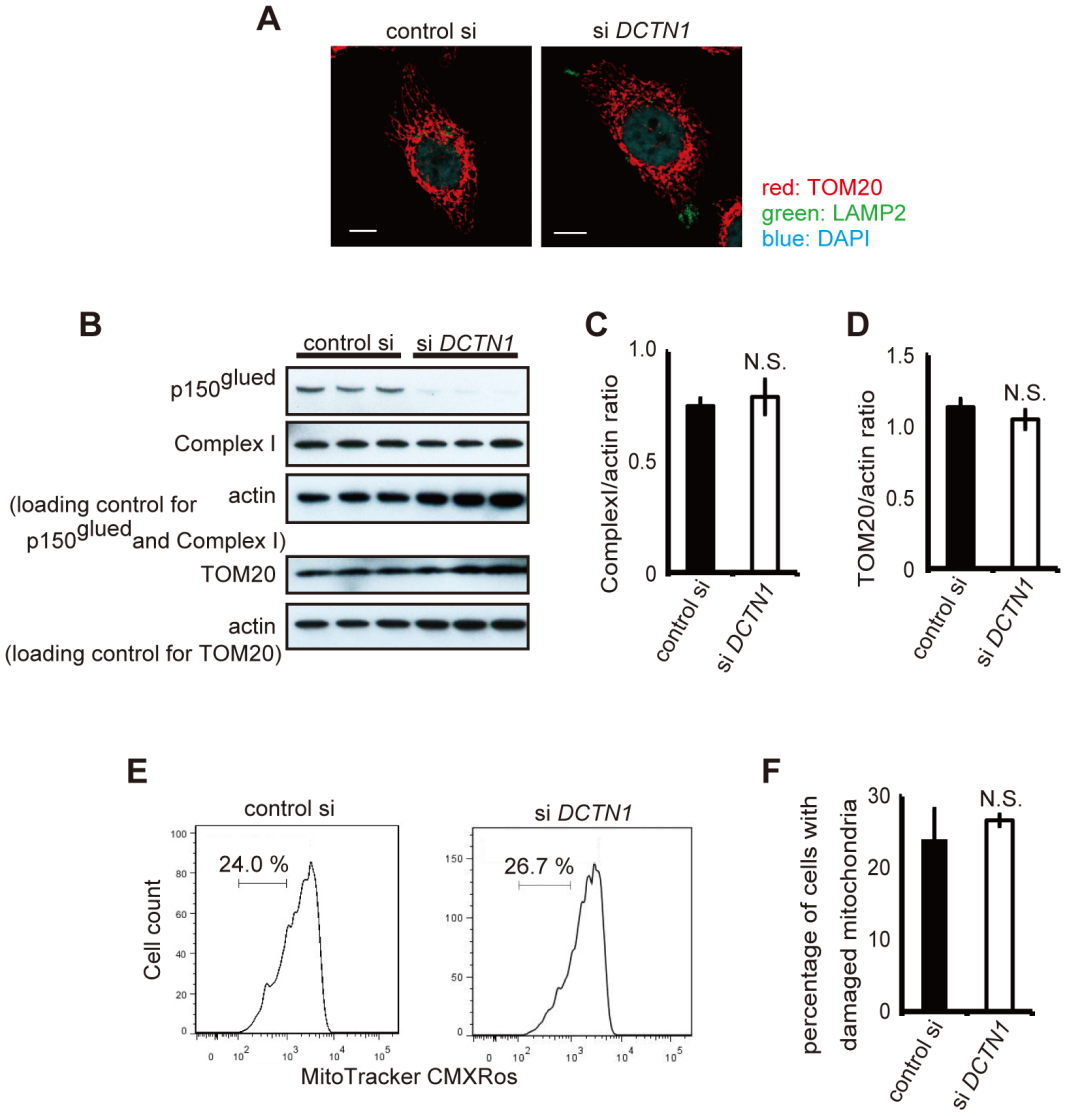
Depletion of p150^glued^ does not induce damaged mitochondria accumulation. (A) Control siRNA or DCTN1 siRNA transfected cells were fixed and co-stained with antibodies against LAMP2 (green) and TOM20 (red), and analyzed using confocal microscopy. Bars, 10 μm. (B) Control siRNA or DCTN1 siRNA transfected HeLa cells were analyzed by immunoblotting with antibodies against complex I, TOM20, and actin. (C, D) Densitometry analysis of complex I (C) and TOM20 (D) levels relative to actin was performed. (E, F) DCTN1 siRNA transfected HeLa cells were incubated with Mitotracker-Red CMXRos and intracellular fluorescence intensity was measured by flow cytometry. The histograms of MitoTracker-Red CMXRos fluorescence (E) and the percentages of cells with reduced mitochondrial potentials (F) are shown. The error bar indicates each standard deviation. Statistics are from three independent experiments: N.S., not significant.

Levy et al. showed that mutant p150^glued^ aggregates are usually associated with mitochondria [11]. Therefore, we hypothesized that an accumulation of damaged mitochondria may cause apoptosis. Live-cell imaging analysis in cells overexpressing WT or mutant p150^glued^ detected elongated tubular mitochondria in control cells, while cells overexpressing mutant p150^glued^ mainly showed fragmented mitochondria in the vicinity of the nuclei (Figure 3A). Overexpression of G59S or G71R p150^glued^ also increased the expression levels of Tom20, a mitochondrial outer membrane protein that is commonly used for assessing mitochondria numbers (Figure 4B, C) [14]–[16].

To determine the health status of the accumulated mitochondria, we next analyzed cells stained with MitoTracker-Red CMXRos by FACS analysis. Only intact mitochondria with preserved respiration activities and membrane potentials absorb this dye. For an accurate assessment, we only analyzed cells that expressed a high GFP intensity, determined using a flow cytometer. Intriguingly, we detected a marked increase in the number of cells with decreased uptake of MitoTracker-Red CMXRos in cells overexpressing G59S or G71R p150^glued^ compared with the control cells (Figure 3D, E). Also, overexpression of WT p150^glued^ decreased mitochondrial membrane potentials, which might be associated with insufficient mitochondria dynamics [11]. Based on the collected results, we conclude that mutated p150^glued^ causes the accumulation of damaged mitochondria, which is followed by activation of the intrinsic apoptotic pathway.

### p150^glued^ knockdown does not affect mitochondrial membrane potentials and activates apoptotic pathway via caspase-8 cleavage

**Figure 5:**
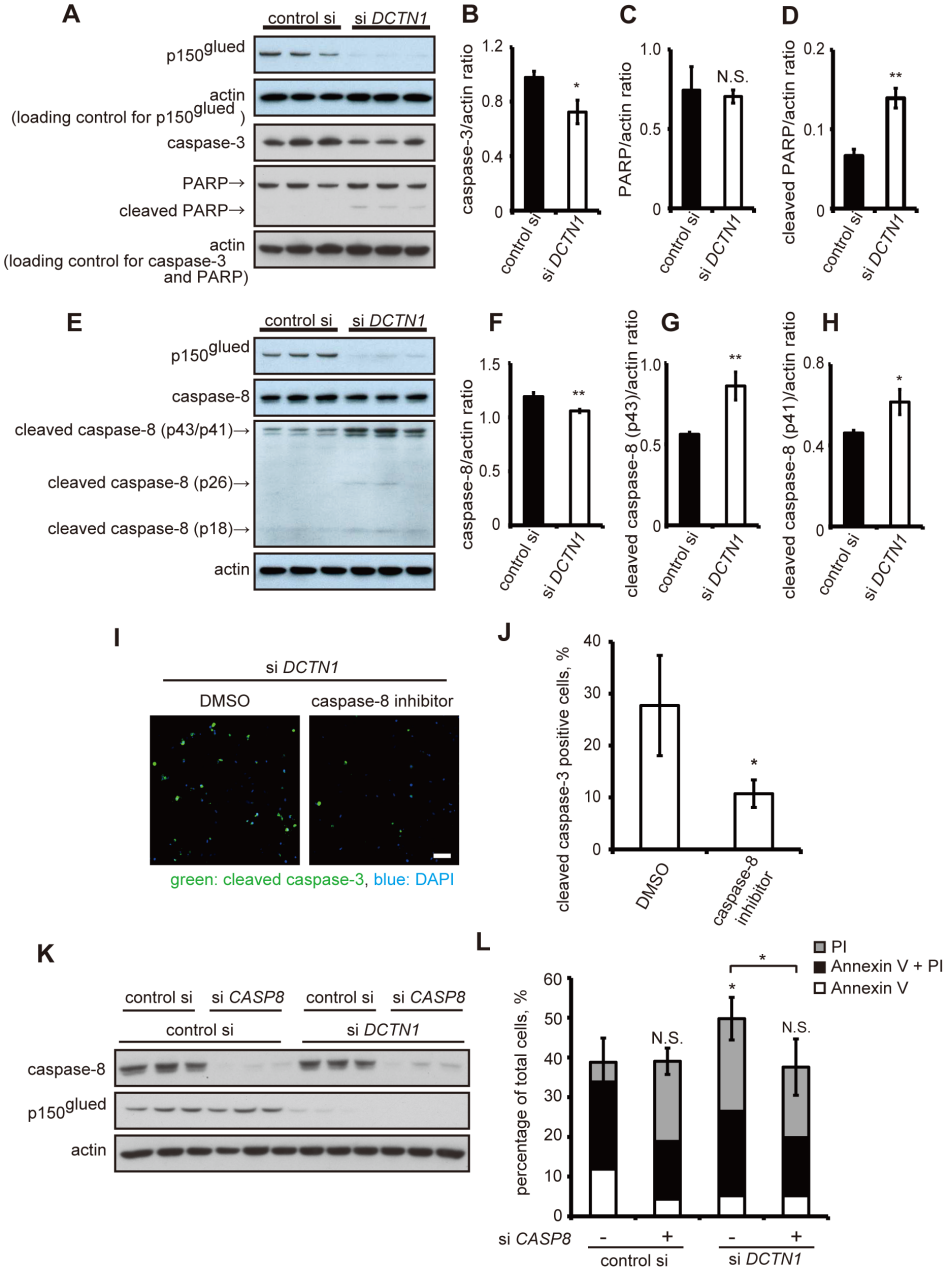
Depletion of p150^glued^ activates apoptotic pathways via caspase-8 cleavage. (A–H) HeLa cells were transfected with control scrambled siRNA or DCTN1 siRNA for 72 h, and immunoblotting analysis was performed with antibodies against caspase-3, PARP, cleaved PARP, and actin (G), or caspase-8, cleaved caspase-8, and actin (E) to monitor the effects on the apoptotic pathway. Densitometry analysis of each protein levels relative to actin was performed (B–D, F–H). (I,J) HeLa cells were transfected with DCTN1 siRNA and incubated with DMSO or 25 μM caspase-8 inhibitor for 48 h. Cells were fixed and stained with antibodies to cleaved caspase-3 (green) and DAPI (blue), and analyzed using fluorescence microscopy (I). The percentage of cleaved caspase-3-positive cells is shown (J). Bars, 100 μm (K,L). Twenty-four hours after transfection with control scrambled siRNA or CASP8 siRNA, HeLa cells were transfected with control or DCTN1 siRNA. Forty-eight hours after DCTN1 siRNA transfection, cells were analyzed by immunoblotting to monitor the knockdown efficiency of caspase-8 and p150^glued^ (K), and stained with Annexin V and PI to assess rates of cell death using flow cytometry (L). The error bar indicates each standard deviation. Statistics are from three independent experiments: N.S., not significant; *,p<0.05; **,p<0.01; ***,p<0.001.

Next, we tested whether or not WT p150^glued^ siRNA knockdown affects mitochondrial functions in a manner similar to mutant p150^glued^ overexpression. As shown in Figure 4A–D, total levels of Tom20 and mitochondria complex I were not changed and the levels of damaged mitochondria without MitoTracker-Red CMX-Ros intake were not significantly increased by p150^glued^ knockdown (Figure 4E, F). Next, we examined caspase-8 activation because of its association with the extrinsic apoptotic pathway. As shown in Figure 5A–H, the levels of total caspase-8 and caspase-3 were decreased with p150^glued^ knockdown, whereas the levels of their cleaved forms of caspase-8 and PARP were increased. This suggests that p150^glued^ knockdown activated caspase-8, leading to caspase-3 activation. Accordingly, treatment with a caspase-8 inhibitor suppressed caspase-3 activation (Figure 5I, J), and caspase-8 siRNA knockdown also decreased apoptotic cell death (Figure 5K, L). Taken together, these data show that the loss-of-function of endogenous p150^glued^ significantly activates caspase-8, inducing apoptosis.

### Cells with mutant p150^glued^ overexpression and wild-type endogenous p150^glued^ knockdown showed more apoptosis

**Figure 6:**
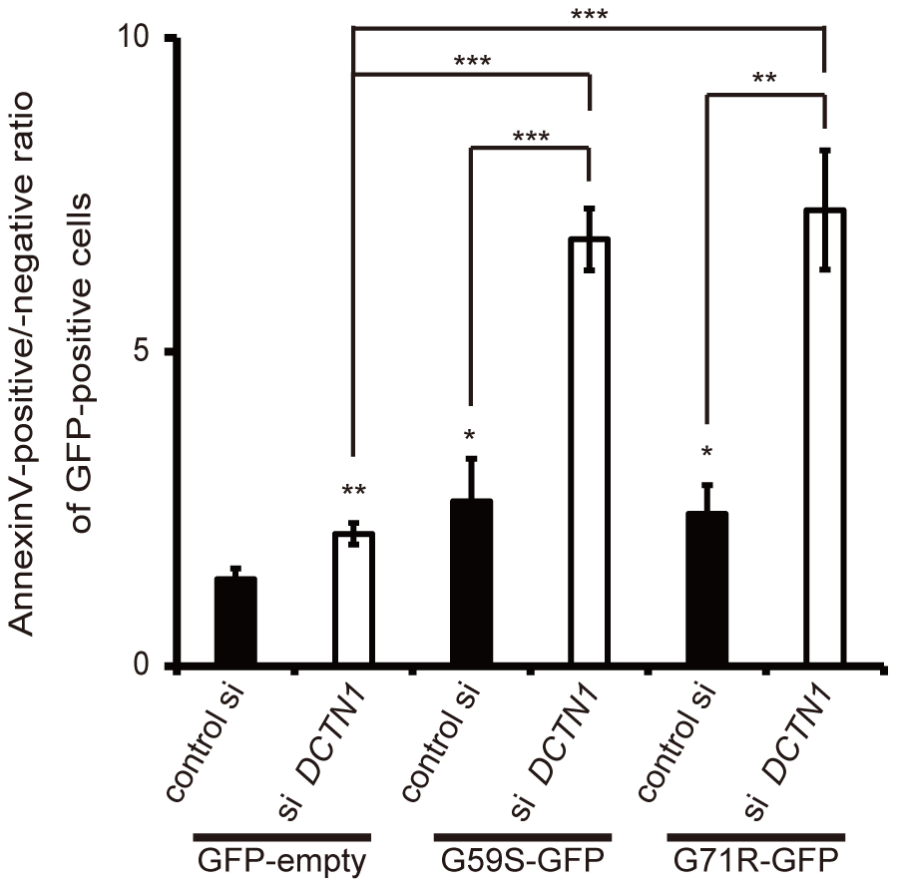
Depletion of p150^glued^ accelerates mutant p150^glued^-induced cell death. Forty-eight hours after transfection with control scrambled siRNA or DCTN1 siRNA, HeLa cells were transfected with GFP-empty vector or mutant (G59S or G71R) p150^glued^ for 24 h. Cells were stained with Annexin V, and GFP-positive cells were analyzed by flow cytometry. The ratios of Annexin V-positive cells relative to Annexin V-negative cells were calculated. The error bar indicates each standard deviation. Statistics are from three independent experiments: *,p<0.05; **,p<0.01; ***,p<0.001.

Finally, to address the pathogenesis of the p150^glued^-associated disorders more precisely, we performed mutant p150^glued^ overexpression experiments with or without siRNA knockdown against endogenous p150^glued^. As shown in Figure 6, siRNA knockdown of endogenous p150^glued^ along with overexpression of either the G59S or G71R mutant form caused many more cells to display early apoptotic changes (GFP-positive and Annexin V-positive) compared with the cells overexpressing a p150^glued^ mutant and a control siRNA. Therefore, we concluded that both excess levels of mutant p150^glued^ and decreased levels of endogenous p150^glued^ contribute to the pathogenesis of p150^glued^-associated disorders via the activation of apoptosis.

## Discussion

In this study, we sought to determine the pathogenesis of p150^glued^-associated diseases caused by p150^glued^ mutations. Overexpression of mutant p150^glued^ in HeLa and SH-SY5Y cells induced p150^glued^-positive aggregate formation, accumulation of damaged mitochondria, and activation of the intrinsic apoptotic pathway. Endogenous p150^glued^ knockdown in the same cell lines also activated a caspase-8-dependent apoptotic pathway without apparent mitochondrial abnormalities. Importantly, cell death induced by p150^glued^ knockdown was markedly enhanced by simultaneous overexpression of mutant p150^glued^, suggesting the disease pathogenesis may be associated with both p150^glued^ gain-of-toxic-function and loss-of-function.

All of the HMN7B and PS associated mutations are located within the CAP-Gly microtubule domain [8], and various reports have suggested that mutant p150^glued^ proteins have the tendency to lose their affinity to microtubules [4], [8]–[10]. Our studies support these reports as we observed decreased colocalization of mutant p150^glued^ with microtubules as well as increased intracytoplasmic aggregates in our immunocytochemistry experiments. However, the *in vivo* binding activity changes of mutant p150^glued^ remain unclear. Therefore, further studies should be performed to determine precisely how mutant p150^glued^ proteins are detoured from their original distribution pattern and how they form aggregates.

The dynein complex plays various critical roles in mitochondrial function (such as retrograde transport and fission/fusion) [17]–[21]. In this study, we could detect mitochondrial abnormalities (loss of membrane potential and morphological abnormalities) only in cells that expressed mutant forms of p150^glued^. A report by Varadi et al. found that disruption of dynein function by either p50 overexpression or microinjection of anti-dynein intermediate chain antibodies led to mitochondrial morphology and distribution changes [22]. Our data, however, showed that p150^glued^ siRNA knockdown did not induce mitochondrial abnormalities. Only mutant p150^glued^ overexpression led to these abnormalities, implying that each dynein subunit might have a specific association with mitochondrial function.

Abnormal protein accumulation has been implicated in the pathogenesis of various neurodegenerative diseases [23]. In this study, we revealed that mutant p150^glued^ (G59S, G71R) overexpression induced aggregate formation and caspase activation associated with mitochondrial abnormalities. This supports the findings by Levy et al. who reported that aggregate formation by the G59S mutant leads to cell death, and that both aggregate formation and the induced cell death were inhibited by overexpression of Hsp70, a molecular chaperone [11]. Likewise, other studies have shown that overexpression of a p150^glued^ plasmid with a truncated C-terminal as well as knockdown of endogenous p150^glued^ in rat hippocampal neurons induces caspase-3-positive cell death, which is consistent with our p150^glued^ knockdown results [24], [25].

According to studies with *in vivo* models, neither expression of a mutant nor the ΔCAP-Gly domain of p150^glued^ affects axonal transport, but WT p150^glued^ is needed for initiation of retrograde transport at synaptic termini in Drosophila motor neurons and mouse dorsal root ganglion neurons [3], [4]. Knock-in and transgenic mice that are heterozygous for the G59S p150^glued^ mutation exhibit late-onset slowly progressive muscle weakness, motor neuron death, and vesicle accumulation [5]–[7]. This is in contrast to heterozygous p150^glued^ knockout mice, which did not display a neurodegenerative phenotype. Taken together, this evidence suggests that the pathogenesis of p150^glued^-associated diseases might be caused mainly by a gain-of-function effect [6], [26].

Neurons of the hypoglossal nucleus and ventral horn in HMN7B and the substantia nigra and locus coeruleus in PS are substantially affected [1], [2]. Although this means that neuronal cell lines are the most appropriate for studying the mechanisms that underlie the pathology of p150^glued^-associated diseases, various studies have been performed using non-neuronal cell lines. For example, the CAP-Gly domain of p150^glued^ was needed for proper Golgi morphology in HeLa cells [10] and to activate cell division in Drosophila S2 cells [27]. G59S overexpression induced mitochondria-containing p150^glued^ aggregates and insufficient recovery of Golgi distribution following nocodazole treatment in COS7 cells. Overexpression of this mutant also increased cell death induction in MN1 mouse embryonic motor neuron cells [11]; however, this overexpression did not promote apoptosis induction by caspase-3 cleavage in COS7 cells or in rat primary motor neurons [28]. Although our data are from non-polarized and non-neuronal HeLa cells and thus might not reflect the precise physiological state of neurodegenerative disease, we believe that these results give us at least partial insight into the mechanisms underlying p150^glued^-associated diseases. Further assessment with more appropriate cell lines, like neurons differentiated from induced pluripotent stem cells from the disease patients should be performed in the future.

## Materials and Methods

### Cell culture and transfection

HeLa and SH-SY5Y cells were maintained as previously described [29]. Cells were transfected using Lipofectamine 2000 (Invitrogen, Carlsbad, CA, USA) according to the manufacturer's protocols. For pharmacological studies, z-VAD-FMK (Calbiochem, San Diego, CA, USA, 219007), caspase-8 inhibitor II (Millipore, Billerica, MA, USA, 218759), and DMSO (Sigma, St. Louis, MO, USA, D2650) were added at the indicated times and concentrations.

### Plasmids

The wild-type *DCTN1* coding region was PCR-amplified from a cDNA plasmid kindly provided by Dr. Farrer MJ (University of British Columbia) using the following primers (Sigma): 5′-TCAAGGGAATTCAATGGCACAGAGCAAGAGGCAC-3′ and 5′-TCAAGGGATATCAGGGAGATGAGGCGACTGTGAA-3′. The resulting fragment was inserted into the pFLAG-CMV5a vector (Sigma) using *Eco*RV and *Eco*RI. The plasmid was cut with *Eco*RI and *Kpn*I, and the insert was subcloned into pAcGFP-N3 (Clontech, Mountain View, CA, USA). Mutagenesis to create the six mutated p150^glued^ plasmids was performed using the Quikchange Lightning site-directed mutagenesis kit (Stratagene, La Jolla, CA, USA). The pCIneo-TDP43-FLAG plasmid was kindly provided by Dr. Koji Yamanaka (RIKEN, Brain Science Institute, Wako, Japan).

### RNA interference

*CASP8*, *CASP3*, and *DCTN1* siRNA were obtained as ON-TARGETplus *CASP8* siRNA SMARTpool (Dharmacon, Lafayette, CO, USA, L-003466-00), ON-TARGETplus *CASP3* siRNA SMARTpool (Dharmacon, L-004307-00), and ON-TARGETplus *DCTN1* siRNA (Dharmacon, J-012874-06), respectively. Non-targeting controls were also purchased from Dharmacon. Transfection of siRNA was performed using Lipofectamine 2000 according to the manufacturer's protocols (Invitrogen).

### Immunocytochemistry

Cells were fixed with 4% paraformaldehyde (PFA) for 15 min, permeabilized with 50 μg/mL digitonin in 1× phosphate-buffered saline (PBS) for 15 min, and incubated with 10% fetal bovine serum (FBS, Invitrogen) and 1% bovine serum albumin (BSA, Wako, Osaka, Japan) in 1× PBS for 30 min at room temperature. Cells were then incubated overnight with primary antibodies at 4°C, followed by incubation with secondary antibodies for 1 h at room temperature. Cells were then mounted with Vectashield containing DAPI (Vector Laboratories, Burlingame, CA, USA). The following antibodies were used in this study: anti-FLAG (Sigma, F7425; 1∶500), anti-α-tubulin (Sigma, T6199; 1∶1000), anti-TOM20 (Santa Cruz Biotechnology, Santa Cruz, CA, USA, sc-11415; 1∶500), and anti-cleaved caspase-3 (Cell Signaling Technology, Danvers, MA, USA, 9661; 1∶800). Alexa Fluor 488- and 594-conjugated secondary antibodies (1∶500) were from Invitrogen. For live-cell imaging, HeLa cells were grown on MatTek glass bottom dishes (MatTek Corp.) and transfected. After 24 hours, 200 nM MitoTracker Red CMXRos (Invitrogen) was added to the media for 15 min at 37°C, followed by a wash in PBS at 37°C. Images were acquired on a Zeiss LSM510 META confocal microscope (Zeiss, Oberkochen, Germany) using a 63× water-immersion objective lens (NA = 1.2). Images were magnified using Zeiss LSM510 v3.2 software. Colocalization was quantified using the colocalization plugin of ImageJ 1.43 (NIH).

### Quantification of aggregate formation and cleaved caspase-3 positive cells

Aggregate formation and cleaved caspase-3 positive cells were assessed using a fluorescence microscope (Axio Imager 2, Zeiss) with a 40× objective. GFP-positive or FLAG-positive cells were selected and the population of cells with aggregates was counted. Quantification was based on at least three independent experiments, each carried out in triplicate, and 100–300 cells were counted in each slide. The scorer was blinded to the identity of the slides.

### Cell viability assays

GFP-positive cells were scored 24 and 48 h after transfection for abnormal cell nuclei, according to previously reported criteria [30] using a fluorescence microscope (Axio Imager 2) with a 40× objective. Analysis was performed with at least three independent experiments, each carried out in triplicate, and 100–400 cells were counted on each slide. The scorer was blinded to the identity of the slides. Detection of apoptotic cells was also determined using an annexin V/propidium iodide (PI) detection kit (Invitrogen), according to the manufacturer's protocol. Briefly, cells were harvested and washed with 1× PBS 24 and 48 h after transfection. They were then incubated at room temperature with annexin V/Alexa350 and PI for 15 min and analyzed by flow cytometry (LSRFortessa, BD Biosciences, San Jose, CA, USA).

### Flow cytometry analysis

Changes in the mitochondrial membrane potentials were assessed with MitoTracker Red CMXRos (Invitrogen). Twenty-four hours after transfection, PBS-washed cells were incubated in 50 nM MitoTracker Red CMXRos for 15 min at 37°C. After washing, cells were suspended in 1× PBS and were analyzed by flow cytometry.

Cleaved caspase-3 labeling was performed according to the flow cytometry protocol from Cell Signaling Technology. Briefly, HeLa cells were pelleted 24 h after transfection, fixed in 4% PFA for 10 min at 37°C and for 1 min on ice. The samples were permeabilized with ice-cold 90% methanol for 30 min on ice and then were blocked in 100 μL of incubation buffer (0.5 g BSA in 100 ml PBS) for 10 min at room temperature. Labeling of the samples was performed with anti-cleaved caspase-3 (Cell Signaling Technology, 9661; 1∶800) antibodies for 1 h at room temperature. After incubation, the samples were washed and resuspended in incubation buffer containing Alexa 647-conjugated secondary antibody (Invitrogen-Molecular Probes; 1∶500) for 30 min at room temperature. The samples were washed, resuspended in PBS and analyzed by flow cytometry. For flow cytometry analysis, 2,000–5,000 GFP-positive cells were analyzed for each sample and the experiments were performed at least in triplicate. Data were analyzed with CellQuest (BD Biosciences) and FlowJo software (Tree Star Inc., Ashland, OR, USA).

### Western blotting

Cells transfected with *DCTN1* siRNA or GFP-tagged vectors for indicated times were lysed in cold RIPA buffer [25 mM Tris-HCl pH 7.6, 150 mM NaCl, 1% NP-40, 1% sodium deoxycholate, 0.1% sodium dodecyl sulfate (SDS)] in the presence of protease inhibitors (Roche, Basel, Switzerland) for 20 min on ice. The lysates were centrifuged and the resulting supernatants mixed in NuPAGE LDS sample buffer (Invitrogen). The samples were resolved on 10–20% Tris-HCl gels (Bio Craft, Agra, India, MDG-297) in 1× Tris/Glycine/SDS buffer (Bio-Rad, Hercules, CA, USA) and transferred onto polyvinylidene fluoride (PVDF) membrane (Millipore). The membranes were blocked for 1 h in Tris-buffered saline (TBS) containing 0.05% Tween-20 (TBS-T) and 5% non-fat milk (BD Difco) and then incubated overnight at 4°C with the primary antibody. The membranes were washed with PBS-T three times followed by incubation for 1 h at room temperature with horseradish peroxidase-conjugated anti-mouse, rabbit, and guinea pig IgG (GE Health Care Biosciences, Pittsburgh, PA, USA). Immunoreactivity was assessed by chemiluminescence reaction using the ECL prime reagent (GE Health Care Biosciences). The antibodies used were as follows: anti-actin (Millipore, clone C4; 1∶10000), anti-p150^glued^ (BD, 610473; 1∶5000), anti-TOM20 (Santa Cruz, sc-11415; 1∶1000), anti-complex I (Invitrogen, 459100; 1∶1000), anti-caspase-3 (Cell Signaling Technology, 9665; 1∶1000), anti-PARP (Cell Signaling Technology, 9542; 1∶1000), anti-caspase-8 (Cell Signaling Technology, 4790; 1∶500), and anti-cleaved caspase-8 (Cell Signaling Technology, 9496; 1∶1000).

### Electron microscopy

HeLa cells were plated on Thermanox plastic coverslips (Nunc, Penfield, NY, USA) and transfected with pAcGFP-empty, wild-type, G59S, and G71R p150^glued^ plasmids. Twenty-four hours after transfection, one set of cells was pre-fixed in 2% glutaraldehyde in 0.1 M phosphate buffer (pH 7.4) at 4°C, and post-fixed with 2% OsO_4_ in phosphate buffer for 1 h at 4°C. After fixation, they were dehydrated in a graded series of ethanol, placed in propylene oxide, and embedded in epoxy resin (Quetol 812, Nisshin-EM, Tokyo, Japan). Ultra-thin sections (90–100 nm) were cut using an ULTRACUT-UCT (Leica, Wetzlar, Germany) with a diamond knife. Sections were stained with 2% uranyl acetate in distilled water for 15 min followed by a lead staining solution for 5 min.

For immune electron microscopic analysis, another set of cells was pre-fixed in 4% PFA and 0.1% glutaraldehyde in phosphate buffer at 4°C, and post-fixed with 1% OsO_4_ and 1.5% potassium ferricyanide in phosphate buffer for 1 h at 4°C. After fixation, they were dehydrated in a graded series of ethanol and embedded in LR White resin. Ultra-thin sections were cut, and samples were incubated in 3.8% sodium periodate for 1 h at room temperature. Samples were blocked with 2% BSA in PBS for 30 min at room temperature and then immunolabeled with primary anti-GFP antibody (Living Colors A.v. Peptide Antibody, Clontech, 632377; 1∶10) followed by anti-rabbit immunogold (BB International, Cardiff, UK; 1∶100). Afterwards, these samples were stained with 2% uranyl acetate in distilled water for 5 min followed by a lead staining solution for 1 min. All sections were examined with a JEM-1200EX (JEOL, Peabody, MA, USA) electron microscope at 80 KV.

### Statistical analysis

Densitometry analysis was performed on immunoblots from three independent experiments using ImageJ 1.43. Differences among means were analyzed using 1- or 2-way ANOVA, followed, when results showed significant differences, by pair-wise comparisons between means using Tukey's Honestly Significant Difference Test. When only two groups were compared, the Student's t test was used. In all analyses, the null hypothesis was rejected at the 0.05 level. SYSTAT 13 software (Hulinks, Tokyo, Japan) was used for statistical calculations.

## Supporting Information

Figure S1 Overexpression of mutant p150^glued^ disrupts p150^glued^ distribution and causes aggregate formation. (A) HeLa cells transfected with GFP-tagged wild-type or mutant p150^glued^ were fixed after 24 h and analyzed using confocal microscopy. Bars, 10 μm. (B) SH-SY5Y cells transfected with GFP-tagged wild-type or mutant (G59S or G71R) p150^glued^ were fixed and stained with an antibody against α-tubulin (red) after 24 h and analyzed using confocal microscopy. Bars, 10 μm. (C) HeLa cells transfected with 3xFLAG-tagged wild-type or mutant p150^glued^ were fixed and co-stained with antibodies against FLAG (green) and α-tubulin (red) after 24 h. Bars, 10 μm. (D) FLAG-positive cells were counted from three independent experiments. The percentage of FLAG-positive cells with aggregates is shown. The error bar indicates each standard deviation. Statistics are from three independent experiments. (E) Electron microscopy examination of HeLa cells transfected with GFP-tagged G59S or G71R p150^glued^. Images on the right are magnified images of the boxed area from the left. Intracytoplasmic aggregate (a) is labeled. (F) HeLa cells were transfected with GFP-tagged wild-type or mutant (G59S or G71R) p150^glued^, and cells were fixed and stained with anti-polyubiquitin antibody (FK2) after 24 h. (G) HeLa cells were co-transfected with FLAG-tagged TDP-43 and GFP-tagged wild-type or mutant (G59S or G71R) p150^glued^, and cells were fixed and stained with antibody against FLAG after 24 h. Bars, 10 μm.
(TIF)

Figure S2 Mutant p150^glued^-dependent apoptosis is not blocked by caspase-8 siRNA knockdown. (A, B) HeLa cells were transfected with control scrambled siRNA or caspase-8 siRNA for 72 h, and immunoblotting analyses were performed to monitor the knockdown efficiency of caspase-8 siRNA (A). Densitometry analysis of caspase-8 levels relative to actin was performed (B). (C, D). Twenty-four hours after transfection with control siRNA or caspase-8 siRNA, HeLa cells were transfected with GFP-empty or GFP-tagged G59S p150^glued^. Forty-eight hours after transfection, cells were stained with Annexin V and PI, and GFP-positive cells were analyzed by flow cytometry. The error bar indicates each standard deviation. Statistics are from three independent experiments: N.S., not significant; ***,p<0.001.
(TIF)
