# Supplementary figures and images for: p150glued-Associated Disorders Are Caused by Activation of Intrinsic Apoptotic Pathway
Source: PLoS One. 2014 Apr 10;9(4):e94645. doi: 10.1371/journal.pone.0094645 (PMC3983229; doi:10.1371/journal.pone.0094645)

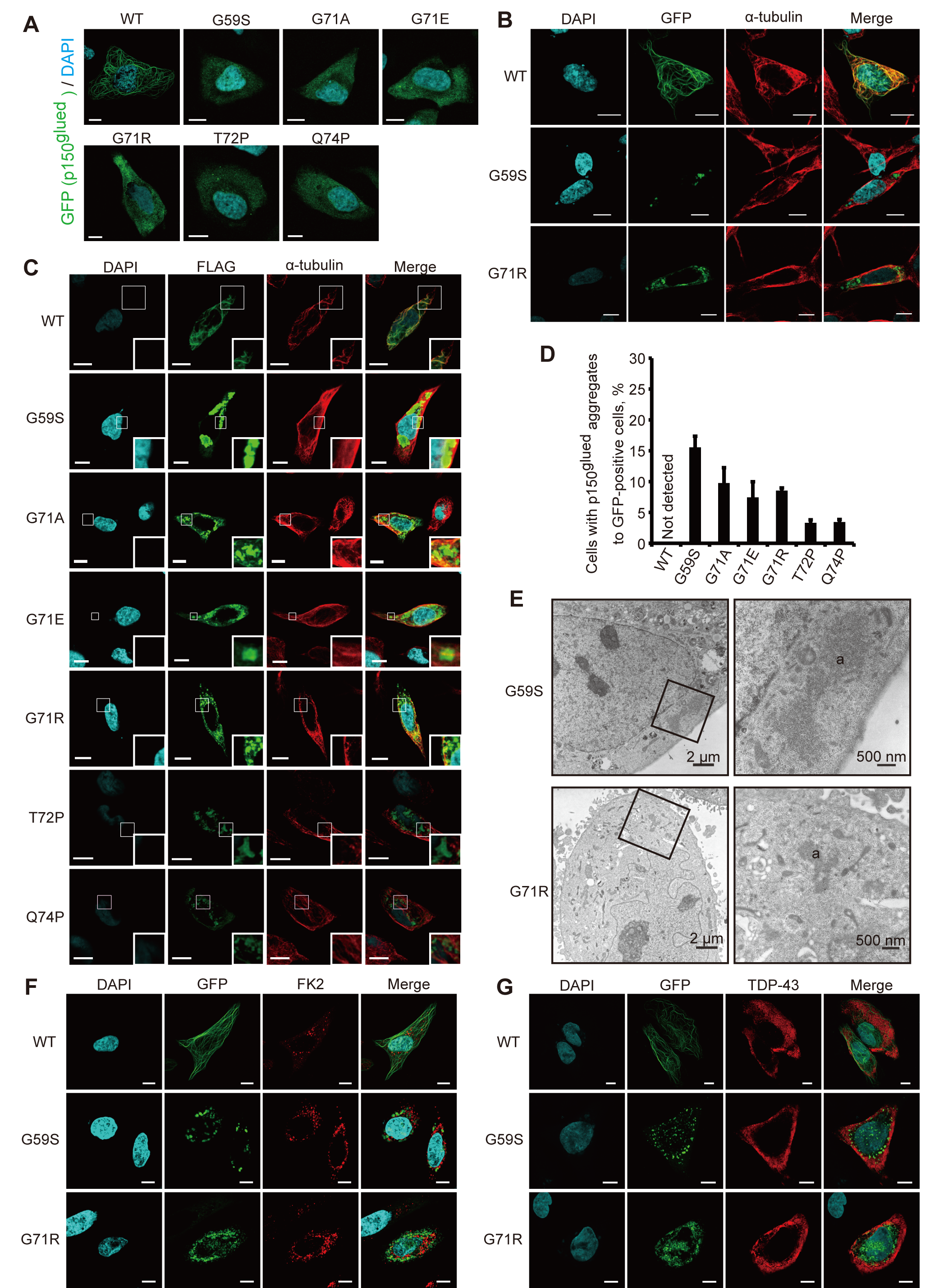

Supplement: Figure S1 — Overexpression of mutant p150glued disrupts p150glued distribution and causes aggregate formation. (A) HeLa cells transfected with GFP-tagged wild-type or mutant p150glued were fixed after 24 h and analyzed using confocal microscopy. Bars, 10 μm. (B) SH-SY5Y cells transfected with GFP-tagged wild-type or mutant (G59S or G71R) p150glued were fixed and stained with an antibody against α-tubulin (red) after 24 h and analyzed using confocal microscopy. Bars, 10 μm. (C) HeLa cells transfected with 3xFLAG-tagged wild-type or mutant p150glued were fixed and co-stained with antibodies against FLAG (green) and α-tubulin (red) after 24 h. Bars, 10 μm. (D) FLAG-positive cells were counted from three independent experiments. The percentage of FLAG-positive cells with aggregates is shown. The error bar indicates each standard deviation. Statistics are from three independent experiments. (E) Electron microscopy examination of HeLa cells transfected with GFP-tagged G59S or G71R p150glued. Images on the right are magnified images of the boxed area from the left. Intracytoplasmic aggregate (a) is labeled. (F) HeLa cells were transfected with GFP-tagged wild-type or mutant (G59S or G71R) p150glued, and cells were fixed and stained with anti-polyubiquitin antibody (FK2) after 24 h. (G) HeLa cells were co-transfected with FLAG-tagged TDP-43 and GFP-tagged wild-type or mutant (G59S or G71R) p150glued, and cells were fixed and stained with antibody against FLAG after 24 h. Bars, 10 μm. (TIF) [file pone.0094645.s001.tif]

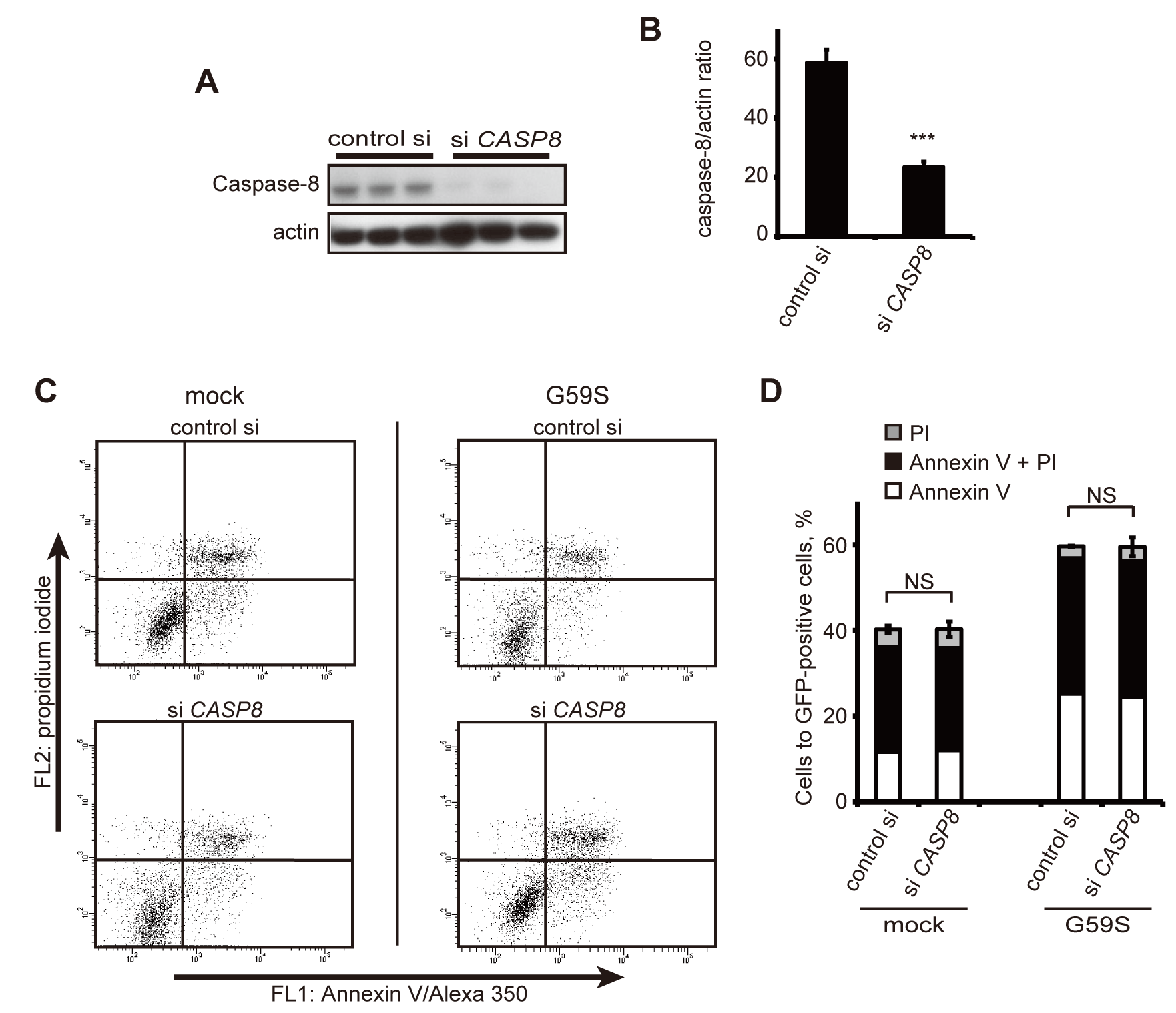

Supplement: Figure S2 — Mutant p150glued-dependent apoptosis is not blocked by caspase-8 siRNA knockdown. (A, B) HeLa cells were transfected with control scrambled siRNA or caspase-8 siRNA for 72 h, and immunoblotting analyses were performed to monitor the knockdown efficiency of caspase-8 siRNA (A). Densitometry analysis of caspase-8 levels relative to actin was performed (B). (C, D). Twenty-four hours after transfection with control siRNA or caspase-8 siRNA, HeLa cells were transfected with GFP-empty or GFP-tagged G59S p150glued. Forty-eight hours after transfection, cells were stained with Annexin V and PI, and GFP-positive cells were analyzed by flow cytometry. The error bar indicates each standard deviation. Statistics are from three independent experiments: N.S., not significant; ***,p<0.001. (TIF) [file pone.0094645.s002.tif]
